# The availability of essential medicines for cardiovascular diseases at healthcare facilities in low- and middle-income countries: The case of Bangladesh

**DOI:** 10.1371/journal.pgph.0001154

**Published:** 2022-11-28

**Authors:** Shariful Hakim, Muhammad Abdul Baker Chowdhury, Md. Ashiqul Haque, Nasar U. Ahmed, Gowranga Kumar Paul, Md. Jamal Uddin

**Affiliations:** 1 Department of Statistics, Shahjalal University of Science & Technology, Sylhet, Bangladesh; 2 Chander Hat Degree College, Nilphamari, Bangladesh; 3 Department of Neurosurgery, College of Medicine, University of Florida, Gainesville, Florida, United States of America; 4 Department of Community Health Sciences, University of Manitoba, Winnipeg, Canada; 5 Department of Epidemiology, Robert Stempel College of Public Health, Florida International University, Miami, Florida, United States of America; 6 Department of Statistics, Mawlana Bhashani Science and Technology University, Santosh, Tangail, Bangladesh; 7 Department of General Educational and Development, Daffodil International University, Dhaka, Bangladesh; PLOS: Public Library of Science, UNITED STATES

## Abstract

Long-term, often lifelong care for cardiovascular disease (CVD) patients requires consistent use of medicine; hence, the availability of essential medicine for CVD (EM-CVD) is vital for treatment, quality of life, and survival. We aimed to assess the availability of EM-CVD and explore healthcare facility (HCF) characteristics associated with the availability of those medicines in Bangladesh. This study utilized publicly available cross-sectional data from the 2014 and 2017 waves of the Bangladesh Health Facilities Survey (BHFS). The analysis included 204 facilities (84 from the 2014 BHFS and 120 from the 2017 BHFS) that provide CVD diagnosis and treatment services. The outcome variable "EM-CVD availability" was calculated as a counting score of the following tracer medicines: angiotensin-converting enzyme (ACE) inhibitors (enalapril), thiazide, beta-blockers (atenolol), calcium channel blockers (amlodipine and nifedipine), aspirin, and simvastatin/atorvastatin. A multivariable Poisson regression model was used to identify the HCF characteristics associated with EM-CVD availability. The number of Bangladeshi HCFs that provide CVD screening and treatment services increased just a little between 2014 and 2017 (from 5.4% to 7.9%). Since 2014, there has been an increase in the availability of calcium channel blockers (from 37.5% to 38.5%), aspirin (from 25.3% to 27.9%), and simvastatin/atorvastatin (from 8.0% to 30.7%), whereas there has been a decrease in the availability of ACE inhibitors (enalapril) (from 12.5% to 6.5%), thiazide (from 15.7% to 11.1%), and beta-blockers (from 42.5% to 32.5%). The likelihood of EM-CVD being available was higher among private and urban facilities than among public and rural facilities. Furthermore, facilities that had 24-hour staff coverage and performed quality assurance activities had a higher chance of having EM-CVD available than those that did not have 24-hour staff coverage and did not undertake quality assurance activities. Government authorities should think about a wide range of policy implications, such as putting more emphasis on public and rural facilities, making sure staff is available 24 hours a day, and performing quality assurance activities at facilities to make EM-CVD more available.

## Introduction

The 2030 Agenda for Sustainable Development Goals (SDGs) of the United Nations (UN) intends to reduce premature deaths from non-communicable diseases (NCDs) by 2030 [1]. Cardiovascular diseases (CVDs) are the most frequent causes of NCDs deaths, accounting for approximately 17.9 million deaths per year, representing 32% of all deaths. Heart attacks and strokes account for over 4 out of every 5 CVD deaths, and a third of such deaths happen prematurely in those younger than the age of 70. If the current trend continues, it is predicted that it will reach 23.3 million by 2030 [2]. CVDs also place a significant financial burden on the patient, the healthcare system, and the nation’s economy. By 2030, global spending on CVD health is forecasted to exceed $1,044 billion, up from $863 billion in 2010 [3].

While the growth in CVD prevalence is a global phenomenon, it has been quickest in low- and middle-income countries (LMICs) over the previous decade. The burden of CVDs continues excessively in LMICs at a greater rate than in high-income countries (HICs), as over 75% of CVD deaths happen in LMICs [2]. Between 2011 and 2025, an estimated cumulative economic loss from all NCDs will be $7.28 trillion in LMICs, and CVDs will be responsible for about half of this estimated loss [4].

Bangladesh, an LMIC, continues to have a high disease burden even with the shift from infectious diseases to chronic diseases [5]. One of the most common diseases in Bangladesh is CVD [6]. CVDs alone account for 25.1% of total deaths in Bangladesh, and this estimate will increase to 37.2% by 2030 [7]. Recent research indicated that 15.5% of Bangladeshi aged 40–69 years are susceptible to CVDs [6]. CVDs and their known associates account for 13.4% of lost disability-adjusted life years (DALYs) [8].

CVD prevention necessitates significant governmental initiatives, infrastructure, and system changes, and the creation of a conducive environment and individual behavioral changes, which can be a long-term and challenging task, particularly in low-resource nations like Bangladesh. However, disease control and health maintenance remain the main feasible options for millions of people with CVD for the time being, and this choice requires treating CVD patients with essential medicines.

In Bangladesh, the healthcare system is shared by the governmental and private sectors. In the public sector, there is a lack of medicine stocks, and hence almost all Bangladeshis acquire their essential medicines through direct out-of-pocket (OOP) purchases from the private sector [9]. In 1995, private OOP health spending accounted for 60% of total health spending; in 2014, the figure was 67% [10]. To run health systems effectively, essential medicines should always be available in sufficient quantities to meet the basic healthcare needs of the population [11]. Access to essential medicines and vaccines that are safe, effective, of high quality, and affordable for all is one of the SDGs [12].

According to Penchansky and Thomas, the idea of access subsumes five dimensions: availability, accessibility, accommodation, acceptability, and affordability [13]. Availability is not only an essential first step, but it is also a challenging aspect, mainly in poorly funded public health systems [14]. A central component of universal health coverage is the continuous availability of essential medicines in the delivery of standard health services [15]. To figure out what to do next, like how to get access to essential medicines for CVD (EM-CVD), it is important to study the availability of the EM-CVD and the challenges they pose for health care systems. Long-term, often lifelong care for CVD patients requires consistent use of medication. Hence, the availability EM-CVD of is vital for treatment, quality of life, and survival.

In 2010, a study investigated the availability of five cardiovascular medicines in 36 countries and described a mean availability of 26.3% in the public sector and 57.3% in the private sector [16]. Prior research in 35 LMICs discovered the mean availability of anti-hypertensive generics at the lowest price of 34.7% in the public sector and 57.1% in the private sector [17]. According to Khatib et al. recent analysis of the availability and affordability of four CVD medicines in pharmacies collected from nearly 600 communities in 18 countries, availability of all four medicines in the urban community was 95 percent, 80 percent, 62 percent, and 25 percent in high-income, upper-middle-income, and low-income countries, respectively [18]. The percentages in the rural community were 90 percent, 73 percent, 37 percent, and 3 percent, respectively. In the West Region of Cameroon in 2014, a study by Jingi et al. found that the availability of drugs for CVD and diabetes ranged from 36.4 percent to 59.1 percent in urban settings and from 9.1 percent to 50 percent in rural settings [19]. In a 2019 study, Kaiser et al. investigated the availability of essential diabetes and CVD medicines, discovering that two anti-diabetics and nine anti-hypertensives had high availability (80%), while the remaining anti-diabetics and anti-hypertensives had availability levels that were mostly under 50% for the rest of the surveyed anti-diabetics and anti-hypertensives in three Zambian provinces [20].

Using data from the 2014–2015 Tanzania Service Provision Assessment (SPA) survey, Bintabara and Ngajilo looked at how ready healthcare facilities (HCFs) were to offer outpatient care for NCDs. They observed that the private higher-level facilities had significantly more basic diagnostic tools and medicines available than their public counterparts [21]. A study by Armstrong et al. revealed that facility region, facility type, managing authority, and range of HIV services are significant correlates of NCD’s medicine availability utilizing 196 Ugandan HCF data [22]. Another study that used the 2015 Nepal SPA showed that private and urban facilities were more prepared to offer services for CVD and diabetes than public facilities and facilities in rural areas [23].

A study by Biswas et al. investigated the preparedness of HCFs for diabetes and cardiovascular services in Bangladesh using the 2014 Bangladesh Health Facility Survey (BHFS) [24]. This study mentioned that among the facilities that offer CVD services, only 0.9% had all four of the four service preparedness factors (guidelines, trained staff, equipment, and medicine) in Bangladesh. To our knowledge, no research has attempted to thoroughly investigate the availability of EM-CVD in Bangladesh to find the HCF characteristics linked with essential medicine availability. We need a deeper understanding of the factors impacting the availability of EM-CVD at HCFs in Bangladesh to accomplish the SDGs by assuring a consistent supply of those medicines. The present study sought to examine the availability of EM-CVD and its associated factors in Bangladesh.

## Materials and methods

### Study population and setting

This study included data from the latest survey rounds of the 2014 and 2017 Bangladesh Health Facilities Survey (BHFS). The BHFS was conducted in 2014 and 2017 by the U.S. Agency for International Development (USAID) and used standardized questionnaires from the service provision assessment (SPA) component of the DHS Program. The National Institute of Population Research and Training (NIPORT) and the Ministry of Health and Family Welfare (MOHFW) ran the survey with help from the Bangladesh government and the United States Agency for International Development. ICF International and the Associates for Community and Population Research (ACPR) collected the data. The Demographic and Health Survey (DHS) runs the survey in collaboration with Bangladesh by the National Institute of Population Research and Training (NIPORT) with technical assistance provided by ICF, USA. The surveys employed identical questionnaires for both the facility inventory and the interviews with the health providers.

The samples for the 2014 and 2017 BHFS were designed to include establishments from across the country’s administrative divisions. The sampling frames were a list of 19,184 and 19,811 registered HCFs from the 2014 and 2017 BHFS, respectively, furnished by NIPORT and MOHFW.

From the complete formal-sector health facilities, 1,596 health facilities for the 2014 BHFS and 1,600 health facilities for the 2017 BHFS were selected using stratified random sampling. Interviewers were unable to survey some of the facilities because they were not open or operating at the time of the survey. Finally, the 2014 and 2017 BHFS contain information on 1,548 and 1,524 facilities, respectively. The sampling strategy and study design are detailed elsewhere [25, 26].

### Selection of study samples

**Fig 1:**
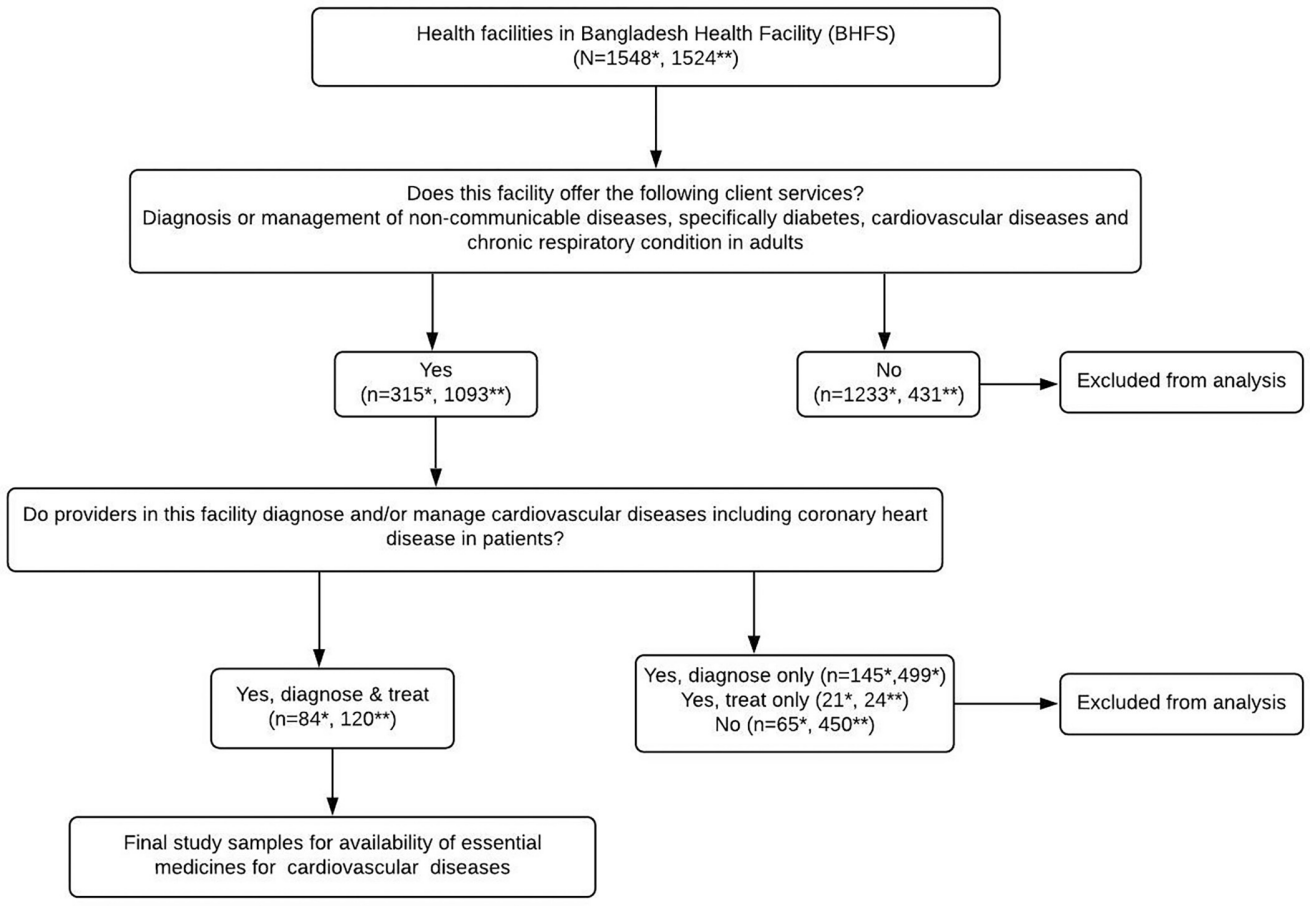
Flow chart of study samples selection. The star (stars) in the figure is a sign indicating the number of healthcare facilities in the 2014 and 2017 BHFS, respectively. One star indicates the facilities from the 2014 survey, and the sign with two stars indicates the facilities from the 2017 survey. Numbers in the parentheses represent weighted data.

Of all the facilities, only those that offer NCD services-especially those where providers diagnose and treat CVD-were included. From the 2014 BHFS, 84 facilities were selected for the availability of EM-CVD, and 120 facilities were included as study samples for the availability of EM-CVD from the 2017 BHFS. The screening process used to select a study sample is illustrated in **Fig 1**.

### Measures

#### Outcome variable

According to the WHO-Service Availability and Readiness Assessment (SARA) reference document [24], the availability of EM-CVD was assessed using a list of tracer drugs: ACE inhibitors (enalapril), thiazide, beta-blockers (atenolol), calcium channel blockers (amlodipine/nifedipine), aspirin, and simvastatin/atorvastatin. Availability was marked out as the presence of at least one legitimate tracer medicine in a facility on the day of the visit, which could be seen by the people collecting the data. Essential medicine availability measures the number of non-expired (valid) tracer drugs for CVD in a health facility. The outcome variable ‘EM-CVD availability’ was calculated as a counting score of the tracer medicines ranging from 0 to 6, where higher scores indicated the greater availability of essential medicines. The scores reflect how many of the six EM-CVD a facility had in stock on the survey day. Therefore, the outcome variable quantifies the number of valid essential drugs for CVD in a health facility.

#### Potential associated factors

Potential associated factors of interest include managing authority (public, private), location (urban, rural), administrative division (Barisal, Chittagong, Dhaka, Khulna, Rajshahi, Rangpur, Sylhet, Mymensingh), external supervision (not received, received within the past 6 months, received more than 6 months ago), routine user fee or charges for client service (not available, available), 24-hour staff coverage (not available, available), system to elicit clients’ opinions about the health facility or its service (not available, available), and guidelines for the diagnosis and management of CVD (not available, available).

### Data analysis

With the help of the Chi-square test, we were able to assess the proportion of EM-CVD availability between the groups of several potential associated factors. The multivariable Poisson regression model was used to identify the health facility characteristics associated with EM-CVD availability. Multicollinearity was checked to see if there was any strong association between the potential factors, and it was determined to be absent. All data management and analyses were conducted using Stata 13 (StataCorp, College Station, TX, USA). To account for the complex survey design, we weighted all of our analyses using the weight option in Stata with the sampling weights provided in the dataset. For modeling exercises, we used the "svy" command of Stata to account for the survey design, primary sampling unit, and cluster.

### Ethics statement

Because we used publicly available, de-identified data from online data repositories, our study was exempt from the ethical review. ICF International’s Institutional Review Board (IRB) and the Bangladesh Medical Research Council of the Ministry of Health and Family Welfare (MOH&FW) have evaluated and approved the methodology and questionnaires for DHS surveys, ensuring that the survey complies with US Department of Health and Human Services regulations for the protection of human subjects (45 CFR 46).

## Results

### EM-CVD availability

**Fig 2:**
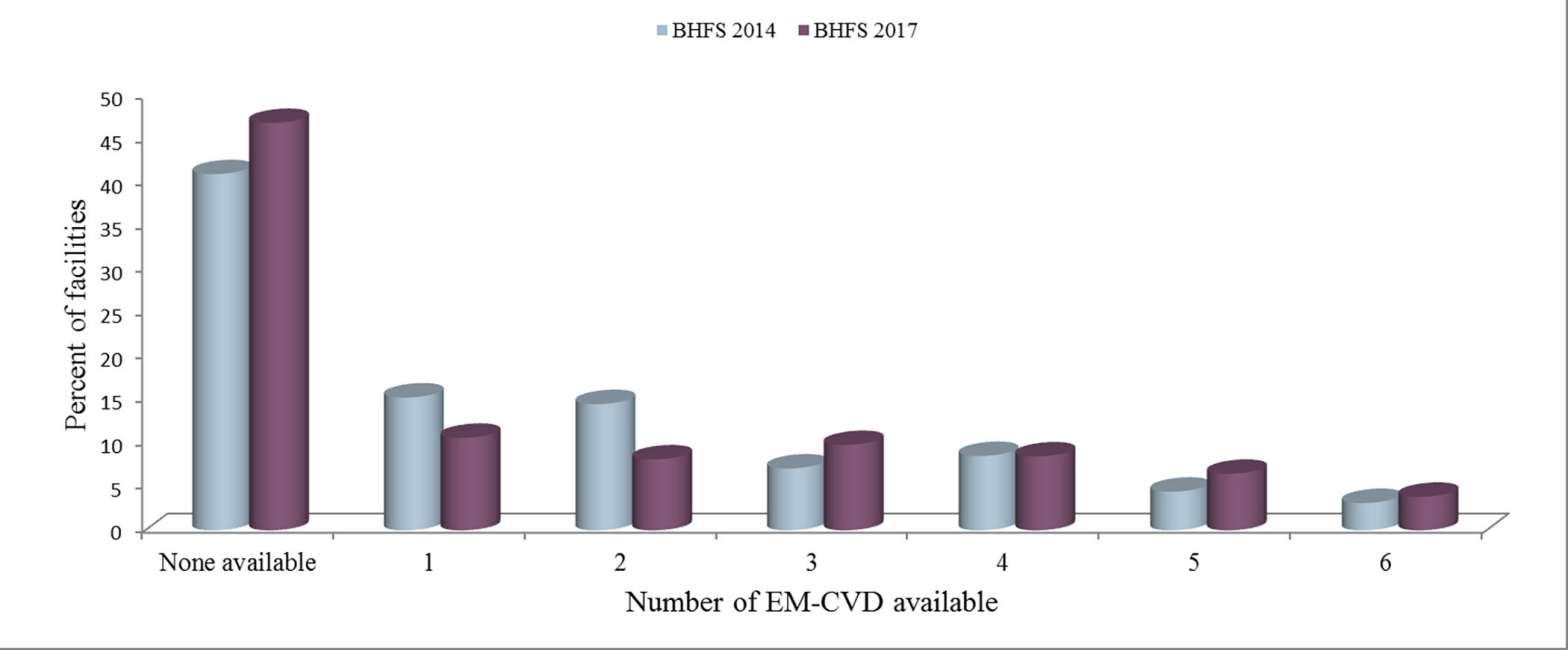
Number of essential medicines for CVD available in sampled facilities, BHFS 2014 and 2017.

The count of various EM-CVD existing at every facility in both the surveys was skewed; scores were gathered at the lowest possible score and with a lengthy tail towards the highest possible score (**Fig 2**). Among facilities offering CVD screening and treatment services, 41.1% (BHFS 2014) and 48.8% (BHFS 2017) of facilities had no EM-CVD on-site at all.

**Fig 3:**
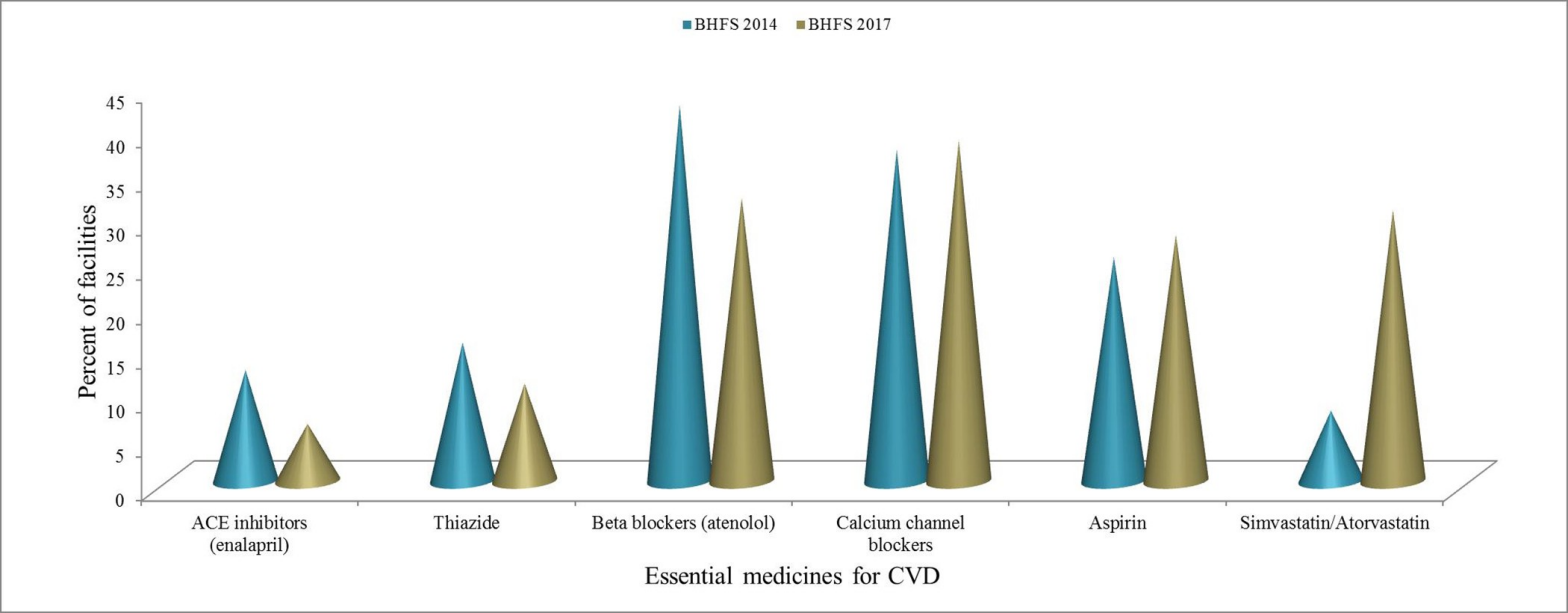
Availability of essential medicines for CVD at health facilities in Bangladesh, BHFS 2014 and 2017.

**Fig 3** summarizes the six EM-CVDs by category and the medicines available at a sample of sites in the 2014 and 2017 BHFS. Between 2014 and 2017, the availability of calcium channel blockers (from 37.5% to 38.5%), aspirin (from 25.3% to 27.8%), and simvastatin/atorvastain (from 8.0% to 30.7%) increased while ACE inhibitors (enalapril) (from 15.7% to 11.1%), thiazide (from 15.7% to 11.1%), and beta-blockers (from 42.5% to 32.1%) fell.

### Background characteristics of surveyed facilities by the number of EM-CVD available

**Table 1** depicts the proportion of EM-CVD availability among the categories of various characteristics of facilities. In 2014, 40.0% of public facilities, 52.3% of rural facilities, and 55.2% of facilities that had no routine user fees or charges for client service did not have any EM-CVD, compared to 44.3%, 56.3%, and 71.6%, respectively, in 2017. Between 2014 and 2017, the availability of four or more EM-CVD decreased among facilities that were situated in Dhaka division (from 30.0% to 22.7%) and that had guidelines for the diagnosis and management of CVD (from 19.7% to 11.1%).

**Table 1:** Distribution of different factors by the number of available essential medicines for CVD, Bangladesh Health Facility Survey (BHFS), 2014 and 2017.

	BHFS 2014 (n = 253)				BHFS 2017 (n = 335)			
Factors	Number of available essential medicines for CVD, n (%)			P-value*	Number of available essential medicines for CVD, n (%)			P-value*
	None available	1–3 available	4 or more available		None available	1–3 available	4 or more available	
**Managing authority**				<0.001				<0.001
Public	68 (40.0)	88 (51.8)	14 (8.2)		105 (44.3)	111 (46.8)	21 (8.9)	
Private	20 (24.1)	33 (39.8)	30 (36.1)		12 (12.1)	39 (39.4)	48 (48.5)	
**Location of facility**				<0.001				<0.001
Urban	42 (25.5)	84 (50.9)	39 (23.6)		46 (21.9)	104 (49.5)	60 (28.6)	
Rural	46 (52.3)	37 (42.0)	5 (5.7)		71 (56.3)	46 (36.5)	9 (7.1)	
**Administrative division**				0.03				0.12
Barishal	9 (22.0)	27 (65.9)	5 (12.2)		15 (30.0)	29 (58)	6 (12.0)	
Chattogram	28 (40.0)	31 (44.3)	11 (15.7)		29 (33.3)	30 (34.5)	28 (32.2)	
Dhaka	15 (25.0)	27 (45.0)	18 (30.0)		15 (34.1)	19 (43.2)	10 (22.7)	
Khulna	7 (29.2)	12 (50.0)	5 (20.8)		15 (41.7)	15 (41.7)	6 (16.7)	
Rajshahi	11 (55.0)	7 (35.0)	2 (10.0)		11 (34.4)	13 (40.6)	8 (25.0)	
Rangpur	6 (37.5)	8 (50.0)	2 (12.5)		6 (20)	17 (56.7)	7 (23.3)	
Sylhet	12 (54.5)	9 (40.9)	1 (4.5)		16 (44.4)	18 (50)	2 (5.6)	
Mymensingh					10 (47.6)	9 (42.9)	2 (9.5)	
**External supervisory visit to facility**				0.31				0.01
Not received	1 (12.5)	2 (25.0)	5 (62.5)		1 (20.0)	0 (0.0)	4 (80.0)	
Received, within the past 6 months	78 (35.5)	106 (48.2)	36 (16.4)		106 (35.7)	139 (46.8)	52 (17.5)	
Received, more than 6 months ago	9 (36.0)	13 (52.0)	3 (12.0)		10 (29.4)	11 (32.4)	13 (38.2)	
**Routine user fee or charges for client service**								<0.001
Not available	53 (55.2)	37 (38.5)	6 (6.3)		63 (71.6)	24 (27.3)	1 (1.1)	
Available	35 (22.3)	84 (53.5)	38 (24.2)		54 (21.8)	126 (50.8)	68 (27.4)	
**24-hours staff coverage**								<0.001
Not available	36 (70.6)	14 (27.5)	1 (2.0)		38 (66.7)	19 (33.3)	0 (0.0)	
Available	52 (25.7)	107 (53.0)	43 (21.3)		79 (28.3)	131 (47.0)	69 (24.7)	
**System to elicit clients’ opinions about the health facility or its services**				0.01				<0.001
Not available	48 (47.1)	38 (37.3)	16 (15.7)		44 (55.7)	23 (29.1)	12 (15.2)	
Available	40 (26.5)	83 (55.0)	28 (18.5)		73 (28.4)	127 (49.4)	57 (22.2)	
**Routine quality assurance activities**				0.01				<0.001
Not performed	40 (50.0)	29 (36.3)	11 (13.8)		53 (58.2)	26 (28.6)	12 (13.2)	
Performed	48 (28.1)	91 (53.2)	32 (18.7)		62 (25.6)	123 (50.8)	57 (23.6)	
**Guideline for the diagnosis and management of cardio-vascular disease**				0.08				0.09
Available	14 (23.0)	35 (57.4)	12 (19.7)		8 (22.2)	24 (66.7)	4 (11.1)	
Not available	74 (38.5)	86 (44.8)	32 (16.7)		109 (36.3)	126 (42.0)	65 (21.7)	

Note: Due to the rounding of cell counts, percentages may not add to 100%. Frequencies and percentages indicate unweighted data.

* P-value for χ2 test.

### Factors associated with EM-CVD availability

**Table 2** indicates the factors associated with the EM-CVD availability using the Poisson regression model. The likelihood of EM-CVD availability scores was 81% more among the private facilities compared to public facilities in 2014 (relative risk (RR): 1.81, confidence interval (CI): 1.25–2.62, p-value: < 0.001). Also, similar findings were found for private facilities compared to public facilities in 2017 (RR: 2.59, CI: 2.02–3.32, p-value: <0.001). The likelihood of EM-CVD availability was 33% lower in rural facilities than in urban sections (RR: 0.67, CI: 0.45–0.98, p-value: 0.04). The same result was also obtained in the 2017 survey (RR: 0.64, CI: 0.46–0.89, p-value: 0.01). When compared to facilities without 24-hour staff coverage, the availability of EM-CVD was 4.11 higher in 2014 (RR: 4.11, CI: 2.11–8.02, p-value: 0.001) and 5.18 higher in 2017 (RR: 5.18, CI: 2.93–9.16, p-value: 0.001). In 2014, facilities from the Sylhet division (RR: 0.44, CI: 0.23–0.85, p-value: 0.02) had lower odds of EM-CVD availability than facilities from other divisions. Moreover, the availability of EM-CVD was higher in the facilities that had routine user fees or charges for client service (RR: 1.67, CI: 1.02, 2.73, p-value: 0.04) and performed routine quality assurance activities (RR: 1.52, CI: 1.11, 2.09, p-value: 0.01) compared to their counterparts.

**Table 2:** The relative risk (RR), 95% confidence interval (CI), and p-values for the availability of essential medicines for CVD obtained using Poisson regression model, the 2014 and 2017 Bangladesh Health Facility Survey (BHFS).

Factors	BHFS 2014		BHFS 2017	
	RR (95% CI)	P-value	RR (95% CI)	P-value
**Managing authority**				
Public	Reference		Reference	
Private	1.81 (1.25, 2.62)	<0.001	2.59 (2.02, 3.32)	<0.001
**Location of facility**				
Urban	Reference		Reference	
Rural	0.67 (0.45, 0.98)	0.04	0.64 (0.46, 0.89)	0.01
**Administrative division**				
Barishal	Reference		Reference	
Chattogram	0.56 (0.35, 0.9)	0.02	0.99 (0.72, 1.36)	0.95
Dhaka	0.66 (0.41, 1.08)	0.10	0.86 (0.57, 1.3)	0.48
Khulna	0.64 (0.37, 1.12)	0.12	0.71 (0.44, 1.15)	0.17
Rajshahi	0.54 (0.22, 1.36)	0.19	0.92 (0.62, 1.36)	0.66
Rangpur	0.52 (0.28, 0.98)	0.04	1 (0.70, 1.44)	0.98
Sylhet	0.44 (0.23, 0.85)	0.02	1.04 (0.68, 1.58)	0.87
Mymensingh			0.75 (0.45, 1.24)	0.25
**External supervisory visit to facility**				
Not received	Reference		Reference	
Received, within the past 6 months	0.88 (0.55, 1.4)	0.59	0.74 (0.49, 1.13)	0.16
Received, more than 6 months ago	0.91 (0.49, 1.67)	0.75	0.82 (0.55, 1.21)	0.31
**Routine user fee or charges for client service**				
Not available	Reference		Reference	
Available	1.17 (0.76, 1.82)	0.47	1.67 (1.02, 2.73)	0.04
**24-hours staff coverage**				
Not available	Reference		Reference	
Available	4.11 (2.11, 8.02)	<0.001	5.18 (2.93, 9.16)	<0.001
**System to elicit clients’ opinions about the health facility or its services**				
Not available	Reference		Reference	
Available	0.96 (0.66, 1.41)	0.85	1.03 (0.78, 1.37)	0.82
**Routine quality assurance activities**				
Not performed	Reference		Reference	
Performed	1.03 (0.71, 1.5)	0.87	1.52 (1.11, 2.09)	0.01
**Guideline for the diagnosis and management of cardio-vascular disease**				
Available	Reference		Reference	
Not available	0.77 (0.54, 1.1)	0.14	0.84 (0.56, 1.27)	0.41

## Discussion

The current study examined the availability of EM-CVD and investigated facility characteristics connected with the availability of these medicines. Between 2014 and 2017, calcium channel blockers and aspirin became more available, while ACE inhibitors (enalapril), thiazides, and beta-blockers became less available. We identified a significant association between EM-CVD availability and managing authority of the facility, facility location, 24-hour staff coverage, and quality assurance activities.

The availability of EM-CVD was low in some countries (e.g., ACE inhibitors: 16.7% in Uganda, 10.0% in Tanzania; thiazide: 4.4% in Nepal, 5.0% in Tanzania; Beta blockers: 20.4% in Uganda, 18.0% in Nepal, 19.0% in Tanzania; calcium channel blockers: 32.7% in Uganda, 11.2% in Nepal, 34.0% in Tanzania; Aspirin: 9.9% in Nepal, 74.0% in Tanzania and statin: 3.1% in Uganda) [21–23] that concur with the findings of this study. The consistency of these studies’ results may be attributable to the similar methodology adopted; both study utilized data from the nationally representative sample obtained by the DHS programme. Consequently, the interview questionnaires were nearly identical. Additionally, these countries have some of the same socio-economic factors because they are both LMICs. In addition, we observed a change in the availability of EM-CVD at HCFs in Bangladesh from 2014 to 2017. The availability of aspirin, calcium channel blockers, and simvastatin/atorvastatin has increased since 2014, while the availability of beta blockers, thiazide, and ACE inhibitors has decreased. The downward trend of the CVD medicines could be caused by a lack of supply, bad stock management, a lack of experienced pharmacists, medicine theft, bad transport and distribution systems, or delayed stock level ordering and monitoring.

Consistent with a prior study [22], our study revealed that private facilities are more likely to have EM-CVD than public facilities. The availability of essential medicine is typically a mandate of the government and it is less available in public facilities frequently due to a poorly funded procurement strategy. However, with several business techniques, the private sector looks to have a considerable influence on the procurement of essential medicines [27]. As a result, these medications are less likely to be available for free at public facilities, but they are readily available at a high cost at private facilities [22]. Furthermore, because private institutions are not subsidized and rely on revenues from clients, they are more interested in providing quality services and meeting clients’ healthcare demands. In doing this, they will be able to develop satisfied and devoted clients who will return to the facility in the future for their requirements and who will also act as a source of referrals for friends and family, thus ensuring the long-term survival of private hospitals [28]. Therefore, the poor availability of essential medicines, specifically in the public sector, needs more concentration and endeavor by the relevant authorities to ensure these medicines are stocked in the facilities [28–31].

Between urban and rural HCFs, previous studies observed significant inequalities in service availability and service provision of health care [29–32]. Consistent with prior studies, the present study found that rural facilities were less likely to have high EM-CVD availability than urban facilities. This finding may be because the healthcare necessities of individuals’ lives in rural areas are different from those in urban areas, and rural areas often experience a lack of access to healthcare. Furthermore, some of the HCFs located in remote and rural areas are affected by little funding and remaining resource constraints, which cause ineptitude in the management of supply logistic systems that result in the poor availability of essential medicines and equipment [33]. Hence, increased efforts are required to draw the attention of policymakers and health service planners focusing on rural HCF to improve CVD medicine availability where most individuals live in rural areas [33].

In 2014, we found that the administrative division of the facility was a strong predictor of the availability of EM-CVD. In Bangladesh, there are distinct divisions where EM-CVD is available. In 2014, facilities from all other divisions had a lower percentage of EM-CVD availability compared to those in Barishal. This may be due to the fact that Barishal had good CVD service provision, making it more probable that these facilities would have EM-CVD. Further research may be done to investigate the underlying causes of this variation among the HCFs in Bangladesh since the reasons of these differences are yet unknown. Additionally, targeted investments are required to enhance service delivery in regions where EM-CVD is less available.

A policy option that numerous developing nations have employed to collect money to fulfill the rising demand for health care services is the partial or full payment for health services by clients [34]. In line with a study [35], we found that the availability of EM-CVD was significantly linked to the facility’s regular user fees, where facilities that charge regular user fees or costs for client services are more likely to have EM-CVD than those that don’t in 2017. User fees are a way for health systems to get more money to improve the quality of health services and offer more services. This could lead to more medicines being available.

Staff scheduling is the method of forming duty timetables for its staff and is a vital portion of staff management of a HCF. Patient satisfaction improves when an HCF’s ability to have excellent personnel on duty at the proper time is an important factor in attending to the patient [36]. Our data reveals that facilities with 24-hour staff scheduling had a higher chance of having EM-CVD than facilities without 24-hour staff scheduling. Facilities with 24-hour personnel coverage may be more likely to provide high-quality services and better meet patients’ needs. As a result, these facilities are more likely to increase service availability, perhaps resulting in high EM-CVD availability. Hence, these facilities are more likely to upgrade the availability of services, which may result in their high availability of EM-CVD.

A process of ensuring and maintaining a high level of service in various HCFs is known as "quality assurance" (QA) [37]. Regular quality assurance activities were significantly associated with EM-CVD availability in 2017, consistent with studies that looked at basic emergency obstetric and newborn care in Tanzania and general services in Bangladesh [38, 39]. Both found that facilities with regular quality assurance activities had better preparedness scores. According to the results of this study, facilities that participated in routine quality assurance activities had a higher chance of having EM-CVD available than facilities that did not participate in quality assurance activities. This is because QA entails continuous monitoring and feedback to improve the delivery of services [40]. Therefore, based on recommendations from the quality assurance team, these facilities have a greater probability of improving the provision of services, which will result in the increased availability of medicines. Despite this, the uptake of quality assurance activities in Bangladeshi health care facilities remains low because only a few facilities conduct routine QA activities and maintain records of these activities [41].

### Strength and limitations

This research has several advantages. To begin with, this is the first study of its kind in Bangladesh, according to our knowledge, and it provides critical insight into the essential medicine availability for CVD patients. The findings of this study, which used a nationally representative sample of HCFs in Bangladesh, give crucial information regarding the factors that influence drug availability for treating CVD. Second, the estimates in this study have been adjusted to account for cluster effects and sample weights because SPA data is acquired using a complex sampling approaches. Finally, we did a comparative analysis utilizing the 2014 and 2017 BHFS to see how changes in EM-CVD availability have changed throughout Bangladesh’s health facilities.

Our approach has some significant drawbacks. To begin with, this study is unable to infer causality. Second, data on essential medicine cost is not collected by the BHFS. As a result, we were unable to directly address the issue of access, which is linked to both availability and cost. Third, BHFS gathered information on how many drugs a particular institution had in stock on a given day. This method is unable to account for changes in supply over time. Fourth, despite a large number of zeros in the outcome variable, we did not use zero-inflated count models in this investigation. Finally, improving NCD outcomes requires taking a close look at prescription pattern and provider behavior. Facilities might not store some of these medications because the healthcare professionals who work there aren’t writing prescriptions for them. We were unable to examine the prescription pattern because these were absent from the health facility surveys.

## Conclusions

For health systems and individuals in Bangladesh and other LMICs that will face the unprecedented burden of CVD in the coming decades, it is crucial to understand the availability of essential medicines and health facility characteristics associated with the availability of these medicines. We found that only a few health facilities possessed adequate necessary medicines for treating CVD. To increase the availability of EM-CVD and to reduce the rising burden of CVDs in LMICs like Bangladesh, relevant authorities should consider a variety of policy options, including a focus on public and rural facilities, 24-hour staff scheduling, and conducting QA activities.
